# Purification and MIC analysis of antimicrobial proteins from *Cucumis sativus* L. seeds

**DOI:** 10.1186/s12906-018-2176-y

**Published:** 2018-04-03

**Authors:** Raid Al Akeel, Ayesha Mateen, Khalid K. Alharbi, Abdullah A. Alyousef, Hazem M. Al-Mandeel, Rabbani Syed

**Affiliations:** 1 Department of Clinical Laboratory Sciences, College of Applied Medical Sciences, King Saud University, Riyadh, 11451, Saudi Arabia; 2 Department of Obstetrics, Gynecology, King Khalid University Hospital & College of Medicine, King Saud University, Riyadh, Saudi Arabia

**Keywords:** Antimicrobial proteins, *Cucumis sativus* L, Ion exchange chromatography

## Abstract

**Background:**

*Cucumis sativus* L. (cucumber), from the family Cucurbitaceae, is a therapeutic plant with various pharmacological benefits, broadly utilized as a part of complementary medicine (e.g., Unani, Ayurveda, Siddha, and Traditional Chinese). In light of past research discoveries, this plant had been chosen to consider its potential antibacterial action.

**Methods:**

Extracts were purified by dialysis and ion exchange chromatography strategy and then assayed for antibacterial activity against four standard pathogenic bacterial strains known to cause foodborne infections and spoilage of food and herbal drugs. Antimicrobial peptides were extracted from seeds using a sodium phosphate citrate (pH 7.2) - CTAB cradle (pH 6.0).

**Results:**

The highest protein concentration was seen with elute fractions 1 and 3 (370 mg/mL) compared with elute fractions 2 and 4 (340 mg/mL). Among the bacteria utilized, *E. coli* was clearly the most sensitive out of selected four strains.

**Conclusion:**

Our results suggest that *Cucumis sativus* L seeds extracts have significant potentials as new antimicrobial agents.

## Background

Antimicrobial peptides (AMPs) are vied as vital constituents of common barriers against attacking pathogens. Typically, they are cationic and amphipathic peptides of variable length and structure. Amid the previous two decades, various AMPs have been identified in a wide range of organisms, including vertebrates, invertebrates and plants [1–4]. Different classes of AMPs are known to be involved in plant resistance against pathogens. Examples include peptides obtained from seeds of *Mirabilis jalapa L.* [5], *Amaranthus caudatus* [6] and *Zea mays* [7], and characterized as being from the thionin family, entities from the lipid exchange proteins (LTPs) family and plant defensins family [8]. Recent studies suggest these peptides are an imperative part of safety against microbial attack [9], and so further study and characterizations of AMPs hold promise for understanding mechanisms of antipathogen defenses.

*Cucumis sativus* L (generic name cucumber), from the family Cucurbitaceae, is an important restorative plant with different pharmacological benefits, generally utilized in traditional drug therapies such as Unani, Ayurveda, Siddha, Traditional Chinese. Previous studies done on the plant demonstrated that the plant has numerous essential phytoconstituents like glycosides, flavones, terpinoids, phytosterol, saponins and anolignan B, tannins, ellargic corrosive, glucose, fructose [10]. These mixes were observed to be involved in a number of the pharmacological exercises, for example, antibacterial, antifungal, antidiabetic, cytotoxic, antacid and carminative movement, hepatoprotective action, and wound healing activities. Explorative research with seed extracts has found potential antifungal and antibacterial activity [11] and thus potentially explains the medicinal basis for this plant. Consequently, this plant had been chosen to contemplate the antibacterial action of protein concentrates from the seeds. Thus far, chromatographic [12, 13] and proteomic approaches [14] have been utilized for peptide discovery. The present study was carried out to extract and purify antimicrobial protein from *Cucumis sativus* L seeds using chromatographic approaches.

## Methods

### Collection of plant material

The *Cucumis sativus* L seeds were obtained from a local market in Riyadh, KSA. Plant seeds were identified by Dr. Ali S Alqahtani, Pharmacognosy Department, King Saud University, Riyadh KSA. Ethical approval was obtained from the research ethics committee (CAMS-153-36/37) in the College of Applied Medical Sciences, King Saud University, Riyadh. A specimen sample of the plant (CS-15116) has been deposited in the Herbarium, Pharmacy College, King Saud University, Riyadh.

### Preparation of plant seed extracts

The extraction and isolation procedures were as per our previous study where we have standardized our protocols for this project [15]. In brief, seed extraction was initiated by first cleaning the seeds with tap water and then distilled water to remove dirt or debris. Seeds were then dried under laminar flow in biological safety cabinet and then ground using a food processor into a fine powder, which was then mixed with sodium phosphate citrate buffer (pH 7.2) and hexadecyltrimethylammonium bromide or cetyltrimethylammounium bromide (CTAB) buffer (pH 6.0) to solubilize proteins. This extraction process was conducted at 28–30^°^C, followed by filtration through Whattmann filter paper No. 1. The filtrate was then mixed 1:1 with a saturated ammonium sulfate solution to precipitate proteins, and then dialyzed utilizing 3 kDa cutoff dialysis tubing (Sigma.UK). Following dialysis, these samples were quantified for protein content spectrophotometrically investigation at 280 nm.

### DEAE-ION exchange chromatography

The chromatography column was prepared using a DEAE cellulose bed of 1 cm thickness, washed with ethanol before use, 20 mM Tris-Hcl for the 250 mL required volume was prepared and pH was attuned to 8.5 using NaOH. Bed preparation: counter ions (salt gradient) of 40 mL of 25 mM NaCl in 50 mM tris (pH -7.2) and 0.3% of DEAE were applied to the column. The elutes were run for 4 h and 2 mL was collected. Each column used a flash chromatography column from Sigma Aldrich, St. Louis, (MO, USA) and the eluting buffer was sodium phosphate citrate buffer (pH -7.2). Elute preparation: four elutes were prepared in test tubes for each dialyzed sample. After dialysis, the samples were poured into the column without disturbing the bed, and left for 20 min to settle. The first eluting buffer, i.e., sodium phosphate citrate buffer (pH -7.2), was loaded into the column and was used to elute the sample. The column was allowed to settle for 15–20 min. The flow rate is adjusted to 1 mL/min and elutes were collected in test tubes at the bottom.

### Bacterial strains

Bacterial strains used for the study were *Staphylococcus aureus*, *Escherichia coli*, *Pseudomonas aeruginosa* and *Proteus vulgaris* obtained from department of Clinical Laboratory Sciences, King Saud University, Riyadh. The cultures were maintained on nutrient agar slants.

### Culture medium and inoculum preparation

High affectability testing agar (Hi-Media) was utilized for checking antibacterial action of seed crude protein concentrates against the test strains. Hi-Media testing agar was blended at a concentration of 23.4 g/1000 ml in distilled water and sterilized by autoclaving. Each test strain was precultured in 10 ml nutrient broth at 37 °C to a cell density of approximately 10^8^ (CFU/mL). All strains were pre-tested to very sensitivity to the positive control antibiotics.

### Agar well diffusion assay

The antibacterial activity of the crude protein concentrates was determined by Hi-Media agar well diffusion as we described previously [16]. Triplicate wells were filled by including 100 μL of crude protein, incubated at 37 °C for 12–24 h, and then inhibition zones measured in millimeters.

### Determination of the minimal inhibitory concentration (MIC)

The protein extract of plant seeds was tested against the reference strains for antibacterial action by micro-dilution method in 96 well microliter plates [17] with minor adjustments and as prescribed by the National Committee for Clinical Laboratory Standard [18]. The suspension of culture with 10^8^cfu/mL concentration added to each well and made a final volume to 200 μL by adding LB broth. Plates were incubated at 37 ± 1 °C for 18 h and after that 10 μL of MTT (5 mg/mL) was added to every well. The plates were analyzed with ELISA plate reader (TECAN) at 530 nm and the lowest concentration of each protein extract which showed complete inhibition was taken as MIC. In control tests, sterile distilled water and ethanol were included in place of protein concentrates; while, antibiotics Chloramphenicol (25mcg) and Ciprofloxacin (100mcg) were utilized as positive controls. For blank reaction, the sterile broth was used in place of suspension cultures (without inoculum).

### Calculations

The percentage inhibition of antibacterial activity of the protein extracts were calculated using the formula: %Inhibition = (A control − Asample) /A control × 100. All analyses were performed in triplicate, with the results expressed as mean ± standard deviation of mean (SEM). The concentration of the protein extract that was required to inhibit 50% of bacterial cell growth (IC_50_) was calculated for different protein seed extracts and was the parameter used to compare the antibacterial activity. A lower IC_50_ means better antibacterial activity.

## Results

### Comparison of protein concentration extracted in different buffers after Dialysis

In our study, two different buffers have been selected to determine the protein concentration in various buffers and pH of protein extracts from *Cucumis sativus* L seeds. Sodium phosphate citrate buffer (pH 7.2) showed good protein extract yield (Table 1) in comparison to the CTAB buffer (pH 6.0).

**Table 1:** Comparison of protein concentration (μg/mL) in *Cucumis sativus* seeds extract by different buffers after dialysis

S.no	Buffers	Protein extract concentration [μg/mL]
1.	Sodium Phosphate Citrate Buffer (pH 7.2)	410
2.	CTAB Buffer (pH 6.0)	370

### Antibacterial activity of crude protein extracts obtained after dialysis

The protein extracts were compared with the standard antibiotic ciprofloxacin [25 μg/ml] and chloramphenicol (100 μg/ml). For both *S. aureus* and *E. coli*, inhibition zones for both types of extracts exceeded that observed with both antibiotics (Table 2). However, this was not the case with *P. aeruginosa* and *P. vulgaris.* Indeed, the seed extracts essentially had no effect on *P. vulgaris*.

**Table 2:** Data of diameter of zone of inhibition of protein extracts from two different buffers after Dialysis

Bacterial Strains	CTAB Buffer (pH 6.0)	Sodium phosphate citrate buffer pH(7.2)	Chl (25mcg/ml)	Cipro (100mcg/ml)
*S.aureus*	28	26	21	16
*E.coli*	13	14	8	14
*P.aeruginosa*	2	13	8	12
*P.vulgaris*	0	5	8	14

### Protein concentration and antimicrobial activity of the ion exchange chromatography eluates

**Fig. 1:**
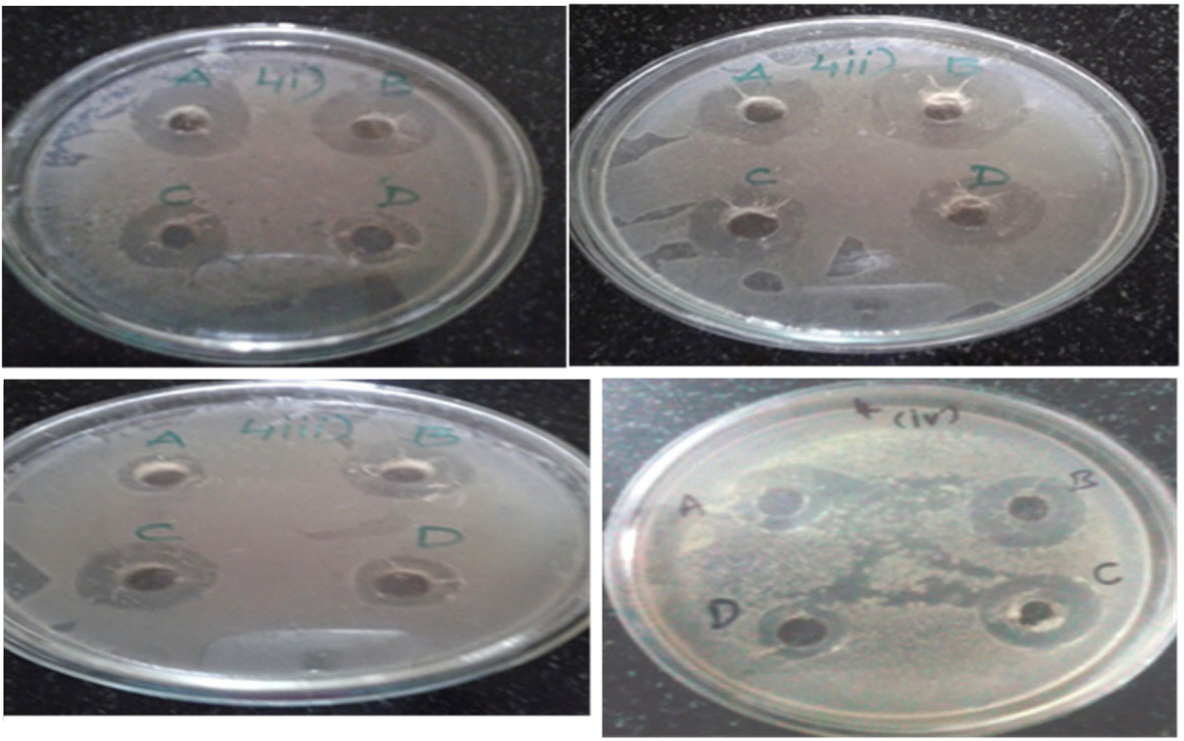
Represents diameter of zone of inhibition of ion exchange chromatography elutes from *Cucumis sativus*.L seeds [i] *S. aureus* (ATCC 25923), [ii] *E. coli* (ATCC 25922), [iii] *P. aeruginosa* (ATCC 27853) and)[iv] *P. vulgaris* (ATCC 6380. Elute 1[A],Elute 2[B], Elute 3[C] and Elute 4[D]

Of the four eluate fractions obtained from the DEAE column, the highest protein concentration was seen with fractions 1 and 3 compared with fractions 2 and 4, although differences were relatively minor (Table 3). Regarding the antimicrobial activity of these extracts, the outcomes are tabulated in (Table 4) and example sensitivity zones are shown in (Fig. 1). Among the bacteria utilized, *E. coli* was clearly the most sensitive. And, the fraction exhibiting the greater antimicrobial activity was seen with elute 2, with 27 mm diameter of zone of inhibition and IC_50_ 121.49 (μg/ml). Indeed, the extract in this fraction proved to be significantly more toxic to *E.coli* than the standard antibiotics. *S. aureus* displayed significant sensitivity to eluate fractions 1 and 2 but essentially no sensitivity to fraction 3. *P. aeruginosa* displayed satisfactory and comparative antibacterial activity with elute 2 and 3 as compared to ciprofloxacin (100 μg), and the relative sensitivity of *P. vulgaris* to all extract fractions was similar to the antibiotics (Table 4).

**Table 3:** Comparison of protein concentration (mcg/mL) after Ion-exchange chromatography

Ion exchange chromatography elutes	Protein concentration
Elute 1	370
Elute 2	340
Elute 3	370
Elute 4	340

**Table 4:** Antibacterial activity of ion exchange chromatography elutes from *Cucumis sativus* seeds, Diameter of zone of inhibition [mm] and IC50 values[mcg/ml]

Bacterial strains	Elute 1		Elute 2		Elute 3		Elute 4		Cipro (100mcg)	
	ZOI	IC50 value	ZOI	IC50 Value	ZOI	IC50 Value	ZOI	IC50 value	ZOI	IC50 Value
*S .aureus*	21	183.53	21	190.28	2	68.15	14	63.78	16	160.529
*E .coli*	26	435.08	27	121.49	21	136.55	24	130.65	14	92.489
*P.aeruginosa*	11	66.45	16	63.63	16	59.97	14	66.9	12	144.634
*P.vulgaris*	18	48.65	17	374.28	15	139.64	17	179.36	14	72.685

## Discussion

There are many research studies showing increased resistance to various drugs in human pathogen and also undesirable effects of certain antimicrobial agents. Therefore, it is important to survey for new proxies that are better, less expensive and without side effect treating infectious diseases, especially in developing countries. A wide assortment of plant/natural products are utilized as a part of the treatment of diseases. Phytoconstituents have been found to showing a promising effect on microorganisms, parasites, viruses and pests [19].

The buffers utilized as a part of the study were sodium phosphate citrate (pH 7.2), which has a buffering zone between pH 5.9–7.9 and CTAB (pH 6.0) which is a nonionic detergent that can precipitate nucleic acids and acidic polysaccharides from low ionic quality solutions [20]. However, proteins and neutral polysaccharides stay in solution under CTAB conditions. Protein concentration varied somewhat as a function of buffer type, but not to a significant degree, indicating that either buffer could be used with this particular seed type. Protein net charge (general negative or positive) depends upon their isoelectric point (pI), and thus their reactivity with the DEAD chromatography matrix [21]. In the present study, the anion exchange chromatography procedure would have selected for negatively charged proteins, influencing which proteins were purified or separated.

In previous studies anionic antimicrobial peptides (AAMP) has been accounted for from an extensive variety of plant species, with net charges that range amongst − 1 and − 7, although the majority are ≤ − 2 [22]. Antibacterial peptides like Cn-AMP2 and Cn-AMP3 each showed a net charge of − 1 and were observed to be dynamic against Gram positive and Gram negative microbes, for example, *S. aureus* and *E. coli* separately, with MICs that were < 250 μM. Another study purified AAMP Cr-ACP that was found to have a net charge of − 1 and shown activity against Gram-positive and Gram-negative microbes [23]. For example, *B. subtilis* and *P. aeruginosa* individually exhibited MICs that were < 65 μM. The present study was in agreement with the past studies, as in the present research work, the protein or peptides extracted were found to be potent against both Gram positive and Gram-negative microorganisms (Table 4). The antimicrobial activity of AAMPs are inadequately understood in spite of the fact that they are known to be capable of targeting intracellular targets like DNA synthesis, or extracellular components such as the cell envelope, degrading cell wall integrity and permeabilization of the plasma membrane via lipid interaction forming pores and channels.

In the present study, protein extraction, purification and antibacterial testing from *Cucumis sativus* L seeds suggests extensive potential for the advancement of new antibacterial agents having potential against diseases that are as of now hard to treat as the bacterial resistance to antibiotics is extending [24], for the treatment of patients. Past studies have demonstrated that asymptomatic bacteriuria presenting more serious hazards in pregnant woman [25], so bacterial resistance is inciting resurgence in research of the antimicrobial part of herbs against resistant strains [26, 27].

Based on our observations, crude and purified proteins extracts from *Cucumis sativus* L seeds could be added to the herbal and food products as additives to upgrade the adequacy of the products. The purified proteins were found to effective on both Gram positive and Gram negative bacterial strains utilized, suggesting this might be a key finding to the creation of new option biodegradable antimicrobial peptide additives, given there is a lack of proficient and safe preservatives in the herbal and food industry. Already reported, the most prominent antibiotic, nisin, is the FDA-approved anti-microbial peptide that has been officially approved as Pharmaceutical preservative [28].

## Conclusion

The present study can be used to discover bioactive natural products in the form of antimicrobial proteins and peptides from *Cucumis sativus* L seeds extract, that may serve for the development of new food preservatives and pharmaceutical product. Further Proteomic analysis in progress to ensure authenticate the results regarding the peptide activity tested in our study.

## Abbreviations

ATCC: American type culture collection
CTAB: Cetyltrimethylammonium bromide
DEAE: Diethylaminoethyl cellulose
IC 50: 50% inhibition
MIC: Minimum inhibitory concentration
MTT: 3-(4,5-Dimethylthiazol-2-yl)-2,5-Diphenyltetrazolium Bromide
pI: Isoelectric point
